# Who aspires to be a scientist/who is allowed in science? Science identity as a lens to exploring the political dimension of the nature of science

**DOI:** 10.1007/s11422-021-10059-3

**Published:** 2021-06-15

**Authors:** Lucy Avraamidou, Renee Schwartz

**Affiliations:** 1 University of Groningen, Groningen, The Netherlands; 2 Georgia State University, Atlanta, USA

**Keywords:** NOS, Science identity, Equity, Pandemic, Reform

## Abstract

Our purpose in this paper is to put forward an argument about both the need and the value for understanding how the constructs of science identity and the nature of science (NOS) might intersect and intertwine and offer useful insights about science participation in times of crises. Based on our knowledge and understanding of these two research areas, we maintain that science identity research has not been fully engaged in understanding how perspectives on NOS might be intersecting with the questions of who can be (or not) a scientist and who is allowed (or not) in science. In this paper, we argue that the formation of a disrupting science identity that challenges existing constructed systems of power in science, requires robust understandings of NOS that place emphasis on the socially-produced narratives about science and scientists. In doing so, we engage with the following questions: (a) How have understandings of NOS contributed to conceptualizations of who can be a scientist and who is recognized as a scientist? (b) How have these conceptualizations contributed to producing exclusionary narratives and perpetuating inequalities in science? and, (c) How might an exploration of NOS through the lens of science identity be used to promote goals related to equity and social justice?

Our purpose in this paper is to put forward an argument about both the need and the value for understanding how the constructs of science identity and the nature of science (NOS) might intersect and offer useful insights about science participation in times of crises. Based on our knowledge and understanding of these two research areas, we maintain that science identity research has not been fully engaged in understanding how perspectives on NOS might be intersecting with the questions of who can be (or not) a scientist and who is allowed (or not) in science. These questions are both crucial and timely, and serve at the heart of the account of re-envisioning the era of the pandemic and reconceptualising the purpose of science education with a focus on goals related to equity and social justice.

Various studies indicate that individuals with minoritized identities, as for example, women and people of color, face scepticism about their intellectual abilities, have undesired identities ascribed to them, and experience misrecognition, racism and sexism as they pursue entrance to the scientific community (Avraamidou, 2020a, b; Carlone and Johnson 2007; Ong, Smith and Ko, 2018). Sociocultural research has shown how the culture of science is aligned with social norms of white, middle-class, heterosexual men (Brickhouse, Lowery and Schultz, 2000; King and Pringle, 2019; Wade-Jaimes and Schwartz, 2019), thus privileging students with these attributes over students considered “other.” The premise of our argument is that in order to address goals related to equity and social justice in science education, it is imperative that the existing constructed systems of power in science, which are framed within specific conceptualizations of NOS, are disrupted.

In what follows, we will argue that the formation of a disrupting science identity that challenges existing constructed systems of power in science, requires robust understandings ofNOS that place emphasis on the socially-produced narratives about science and scientists. These narratives are inextricably bounded within specific sociopolitcal contexts and are directly related to the construct of “recognition”, which, in the context of science education research, refers to the act of recognizing someone as a science person (Avraamidou, 2020a, 2020b). (Mis)recognition is a Bourdieusian concept that has been used in educational research to refer to the relationship between educational success and social advantage or social capital. As Bourdieu stated:

Misrecognition of the social determinants of the education career—and therefore of the social trajectory it helps to determine—gives the educational certificate the value of a natural right and makes the educational system one of the fundamental agencies of the maintenance of the social order. (Bourdieu, 1984, p. 387)

Of interest to us, in this paper, is the attention to “social order” which is used to refer to social inequality. More precisely, the construct of (mis)recognition is tied to two interrelated questions that are rooted within contemporary conceptualizations of science identity and NOS: who aspires to be a scientist and who is allowed in science.

The paper unfolds as follows. We begin by situating our argument within current socio-political realities and global socioscientific challenges. We then discuss briefly the possible role of science education in addressing these challenges. We argue that exploring conceptualizations of NOS, through the lens of science identity, is necessary to gain a sense of if and how learners’ understandings of NOS may facilitate or hinder their development of science identity.In other words, if a person with marginalized identities (e.g., Black, woman, queer) experiences science as a community of white, heterosexual, middle-class men with superior intellect; and these men (aka gatekeepers) produce absolute knowledge in an isolated, culture-free, and methodical way; then how can a science identity develop and compatibly intersect with their racial, ethnic, gendered, cultural identities (among others)?

Toward these goals, we will engage with the following questions:

- How have understandings of NOS contributed to conceptualizations of who can be a scientist and who is recognized as a scientist?
- How have these conceptualizations contributed to producing exclusionary narratives and perpetuating inequalities in science?
- How might an exploration of NOS through the lens of science identity be used to promote goals related to equity and social justice?

## Here and now

Globally, as of 18 April, 2021, there have been more than 140 million confirmed COVID-19 cases while the number of related deaths has reached more than 3 million. At the same time, second waves of infections are reported in various parts of the world, which means that the number of deaths will only continue to rise. As much as a biological threat, the pandemic is also a threat at the societal level. The mismanagement in different countries not only has not contributed toward addressing the disastrous consequences of the pandemic, but it has, in fact, become part of the disaster. Concurrently, we are witnessing racism, Islamophobia, inequalities, poverty, climate change, and the global refugee crisis in different parts of the world. Parallel to these challenges, we witness a rise of anti-science and “alternative facts” movement: pseudoscience, fake news, conspiracy theories and hostile fantasies that undermine scientific evidence. This has led to the post-truth politics which, according to the Oxford English Dictionary, refers to the cherry-picking of data to come to whatever conclusion one desires. Opinions of those in power positions are touted as “truth” simply because these authorities state what they want, repeatedly, in efforts to cast doubt on actual scientific evidence. All this is done at the demise of human safety in order to prop up egos and power. The socio-political climate where we find ourselves is notably controlled by white, male, dominant administrations with xenophobic rhetoric, dangerous calls for social unrest, and scientific illiteracy.

This socio-political climate alongside the rise of anti-science require urgent solutions at different levels; science education is no exception. We maintain that science education has a crucial role to play in addressing goals related to promoting scientific literacy and active scientific citizenship as well as goals related to equity and social justice especially in terms of access to educational resources and opportunities. To achieve these goals, we propose that science education researchers actively engage in efforts to diversify theoretical and epistemological orientations and related methodological choices through an examination of the intersection of the constructs ofNOS and science identity. With the goal of advancing scientific literacy in these times of crises, we consider how science identities develop and how NOS conceptions fit, or not, with science identities. Within these contexts, we ask, who aspires to be a scientist and/or who is allowed in science?

## Science identity

Quite a few researchers in science education have engaged with the construct of “science identity” in the past decade to examine study- and career-choices, science engagement, science learning, pedagogies and teaching practices, as well as lived experiences of students, teachers, and scientists in relation to science. A basic conceptualization of science identity, as put forward by Heidi Carlone and Angela Johnson (2007) refers to how people see themselves (self-view) as science persons, and how they are recognized (viewed by others) as science persons. Self-view and recognition are inextricably bounded and rooted within the question of who aspires to be a scientist and who is allowed in science.

As argued elsewhere, science identity not only provides a valuable lens for exploring science (non)participation but also a lens for better understanding the complexities of becoming a science person which are tied to societal, structural, and political issues (Avraamidou, 2020a). A review of the literature shows that various factors at different levels influence science identity development: individual or interpersonal level, institutional level which refers to schools or universities, the societal level which refers to influences that individuals experience as members of different social communities, and, the political level, which refers to the socio-political realities of specific contexts. It is precisely these socio-political realities that we are interested in exploring and how these might serve as either bridges or barriers to someone aspiring to be a scientist or to someone being allowed in science.

These issues are captured through the construct of recognition and are deeply rooted in as well as affected by conceptualizations of NOS. Most theories of recognition assume that in order to develop a practical identity, persons fundamentally depend on the feedback of other subjects and of society as a whole (Mattias, 2013)—a process that is neither neutral nor apolitical. As a construct, (equal) recognition is of paramount importance to science because it is inextricably bound with cultural, social, and political factors. As Charles Taylor (1992) argued:

Equal recognition is not just the appropriate mode for a healthy democratic society. Its refusal can inflict damage on those who are denied it…the project of an inferior or demeaning image on another can actually distort and oppress, to the extent that the image is internalized. (p. 36)

Hence, recognition points to the political, social, and ethical dimensions of science identity. The question then becomes one of how have understandings of NOS contributed to conceptualizations of who is recognized as a scientist and how have these conceptualizations contributed to producing exclusionary narratives and perpetuating inequalities in science?

## Nature of science

By NOS, we refer to the epistemic features and functions of scientific knowledge. While scholars have offered various depictions of NOS, there is currently general agreement on certain characteristics attributed to scientific knowledge (Lederman and Lederman, 2014). Included among these characteristics are that scientific knowledge is based on observations and inferences of the empirical (natural) world. The knowledge that is developed is dependent upon decisions regarding what data/observations to collect, how to collect, and how to negotiate meaning from data that results in an evidence-based and science community-based claim. Such decisions (and as such, scientific knowledge) are necessarily and productively influenced by not only the scientists, but also the socio-political and cultural climate at the time.

Clearly, in today’s socio-political climate and within the context of the pandemic, understanding NOS has renewed importance and implications. The science is moving quickly; new understandings about COVID-19 and manners to combat, treat, and protect ourselves are advancing almost weekly. These advances and the vocal mistrust of science touted by political authorities are in conflict with each other. Without understanding NOS knowledge as dynamic, empirical, and socioculturally influenced, the general public is left wondering who to believe. Tackling issues of equity and representation in who can be a scientist is further distanced due to the visible political crowds voicing disrespect and doubt on science and scientists. Questions of equity and representation have been explored in science education through the lens of science identity.

## Intersections of NOS and science identity

Research studies provide evidence that in order to access and succeed in a science field, individuals must develop strong science identities and believe they can become productive and meaningful members of the science community (Avraamidou, 2020a, b). However, an examination of related literature shows that who is seen as a “science person” is highly gendered and racialized (Carlone and Johnson, 2007) and the construction of individual science identity is complicated by history and power dynamics (Carlone and Johnson, 2007). The white male hegemonic context and deep-seated gender/race beliefs and barriers in STEM lead to individuals with minoritized identities in science (e.g., women and people of color) being challenged to see themselves as contributing whole members of the scientific community (Archer, DeWitt and Osborne 2015; Nasir, 2011).

Research has shown consistent limitations regarding people’s understandings of NOS (e.g., Erduran & Dagzer, 2014). Public images, curricula, assessments, teaching resources, and the media have consistently portrayed scientific knowledge and the scientific endeavour as absolute “facts” generated through a linear and systemic process (aka the scientific method) that is objective and independent of creativity, culture, values, and social contexts. From decades of research soliciting teachers’ and students’ views of NOS and the qualities that make someone a “science person” or “scientist” indicate over and over again the perception that scientists are male, white, dishevelled loners with elite intellect and a compulsion for mixing bubbly chemicals in a dark and sterile laboratory.

**Fig. 1:**
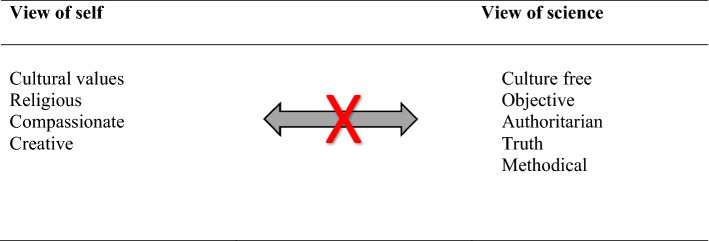
Incompatible view of self and NOS

We argue that this image of science and scientists may serve to, at least partially, explain the underrepresentation of specific groups, as for example, women and people of color in science careers. In other words, the community of science is not welcoming to those who do not “look and act in certain ways.” For example, while a person of color may have the competence and skilled performance of science, if she does not recognize herself and if others do not recognize her as fitting their image of what science is and who can be a scientist, then this person is likely to choose another career path. Figure 1 depicts this type of incompatible relationship between view of NOS and view of self. The view of self may include valuing one’s culture, perhaps including language, traditions, and religious values. View of self may include compassion, care, and creativity. Such identities are then in conflict if the view of NOS is as culture free, objective, and final truth and authority. Such conflict may not be resolved to enable access to science unless the view of self assimilates into that view of NOS. This conflict could also render the individual doubting the relevance of scientific claims. Not only does this situation serve as a barrier to who has access and recognition as a scientist, it perpetuates inequities and false narratives about whose science is valued.

The question becomes one of what can be done? Does an individual have to strip off or shift their identities so they align with and achieve recognition within this cultural hegemony? To that end, the cycle of absolutist, culture and value free NOS knowledge is upheld. Inequities persist. Hegemony begets hegemony. In times of crises, power speaks to power, with scientific literacy pushed aside or misrepresented.

We recommend another approach: Disrupt the incompatible view of NOS to focus on a more holistic, inclusive, and equitable representation of NOS and who can be a scientist that embraces the diversity and subjectivity of identity in general and science in particular. A departure point perhaps would be, as Sibel Erduran, Ebru Kaya and Lucy Avraamidou (2020) argued, to explore the relations between key constructs in contemporary conceptualizations of NOS (e.g., epistemic features of scientific knowledge, social values, political power structures) and social justice (e.g., difference principle, opportunities for fulfilling lives, freedom). Such a holistic approach to NOS promises to bring to the surface the social-institutional dimensions of science which serve as barriers to science identity development.

**Fig. 2:**
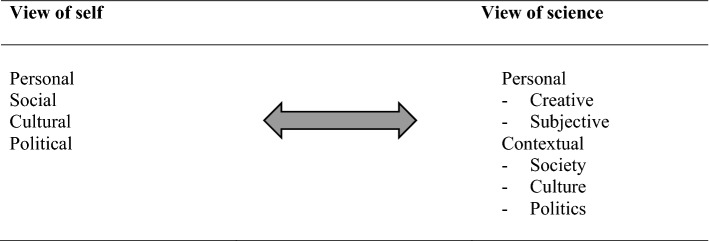
Compatible view of self and NOS

Embracing the cultural influences within science may open access of science to those who have not typically seen themselves or people who look, talk, act, worship like them as productive members and leaders of the scientific community. As Heidi Carlone, Angela Johnson and Margaret Eisenhart (2014) argued, “a cultural lens for science education can be a tool for counter-hegemony” (p. 667). Given that to recognize self, or future self, as a science person, involves identity components at the personal, social, cultural, and political level (Avraamidou, 2020b), then the view of NOS should also recognize that knowledge is embedded within personal and contextual or cultural features (Fig. 2).

## Gazing forward

Despite the key contributions of research in the area of the nature of science on science teaching and learning, researchers have not fully engaged with the politicized NOS or the political, ethical and cultural dimensions of science. This politicized NOS, we argue, might be explored through the lens of science identity. There is still much to be understood about science identity and how might an exploration of NOS through the lens of science identity be used to promote goals related to social justice and equity. We may never be able to fully understand the complexity and multiplicity of science identity and how it develops, but we can begin to challenge deficit understandings of NOS which have contributed to producing exclusionary narratives about science as well as determining the kinds of identities that are allowed in science.

We hope that this paper will serve as a springboard for conversations about the development of critical theoretical frameworks, curricular, out-of-school programs, teacher preparation programs as well as professional development programs at the intersection of NOS and science identity that challenge deficit and exclusionary understandings of what science is and who can do science. An examination of the intersection of NOS and science identity that works from a critical and intersectional standpoint and which aims to support young people develop disruptive identities is not only valuable, but crucial in these times of crises and in re-envisioning the post-pandemic era.

## References

[CR1] Archer L, Dewitt J, Osborne J (2015). Is science for us? Black students’ and parents’ views of science and science careers. Science Education.

[CR2] Avraamidou L (2020). Science identity as a landscape of becoming: Rethinking recognition and emotions through an intersectionality lens. Cultural Studies of Science Education.

[CR3] Avraamidou L (2020). “I am a young immigrant woman doing physics and on top of that I am Muslim”: Identities, intersections, and negotiations. Journal of Research in Science Teaching.

[CR4] Bourdieu P (1984). Distinction: A social critique of the judgement of taste.

[CR5] Brickhouse NW, Lowery P, Schultz K (2000). What kind of a girl does science? The construction of school science identities. Journal of Research in Science Teaching.

[CR6] Carlone HB, Johnson A, Eisenhart M, Lederman NG, Abell SK (2014). Cultural perspectives in science education. Handbook of research on science education.

[CR7] Carlone HB, Johnson A (2007). Understanding the science experiences of successful women of color: Science identity as an analytical lens. Journal of Research in Science Teaching.

[CR8] Erduran S, Kaya E, Avraamidou L, Yacoubian HA, Hansson L (2020). Does research on nature of science and social justice intersect? Exploring theoretical and practical convergence for science education. Nature of science for social justice.

[CR9] Erduran S, Dagzer Z (2014). Reconceptualizing the nature of science for science education: Scientific knowledge, practices and other family categories.

[CR10] King N, Pringle R (2019). Black girls speak STEM: Counterstories of informal and formal learning experiences. Journal of Research in Science Teaching.

[CR11] Lederman NG, Lederman JS, Lederman NG, Abell SK (2014). Research on teaching and learning of nature of science. Handbook of research on science education.

[CR12] Mattias, I. (2013). Recognition. In E. N. Zalta (Ed.). *The Stanford encyclopedia of philosophy*. Retrieved August 27, 2018, from https://plato.stanford.edu/archives/fall2013/entries/recognition/.

[CR13] Nasir NS (2011). Racialized identities: Race and achievement among African American Youth.

[CR14] Ong M, Smith JM, Ko LT (2018). Counterspaces for women of color in STEM higher education: Marginal and central spaces for persistence and success. Journal of Research in Science Teaching.

[CR15] Taylor C (1992). Multiculturalism and the politics of recognition.

[CR16] Wade-Jaimes K, Schwartz R (2019). “I don’t think it’s science:” African American girls and the figured world of school science. Journal of Research in Science Teaching.

